# Comparison of Effectiveness and Safety of Low-Dose Versus Standard-Dose Intravenous Recombinant Tissue Plasminogen Activator in Patients With Acute Ischemic Stroke: A Meta-Analysis

**DOI:** 10.7759/cureus.35571

**Published:** 2023-02-28

**Authors:** Jithin Karedath, FNU Avanteeka, Muhammad Nouman Aslam, Ahmad Nadeem, Rao Ahmed Yousaf, Sandesh Shah, Sujith K Palleti, Areeba Khan

**Affiliations:** 1 Internal Medicine, King's College Hospital NHS Foundation Trust, London, GBR; 2 Internal Medicine, Liaquat University of Medical and Health Sciences, Jamshoro, PAK; 3 Medical College, King Edwards Medical University, Lahore, PAK; 4 Medicine, Liaquat National Hospital, Karachi, PAK; 5 Medical College, Faisalabad Medical University, Faisalabad, PAK; 6 Department of Dermatology, KIST Medical College, Lalitpur, NPL; 7 Nephrology, Edward Hines Jr. Veterans Administration Hospital, Hines, USA; 8 Nephrology, Loyola University Medical Center, Maywood, USA; 9 Critical Care Medicine, United Medical and Dental College, Karachi, PAK

**Keywords:** meta-analysis, recombinant tissue plasminogen activator, standard dose, stroke, low dose

## Abstract

The aim of the present meta-analysis is to compare the efficacy and safety of low-dose and standard-dose recombinant tissue plasminogen activators (r-tPA) in patients with acute ischemic stroke. The present meta-analysis was conducted according to the Meta-Analysis of Observational Studies in Epidemiology (MOOSE) guidelines. We conducted a systematic search in PubMed, Embase, and the Cochrane Library to identify studies published between January 1, 2010, and January 31, 2023, using the following terms: "stroke," "alteplase," "doses," "efficacy," "tissue plasminogen activator," "r-tPA," and "safety." Primary efficacy outcomes included favorable outcomes (Modified Rankin Scale scores of 0-2), while secondary efficacy outcome was all-cause mortality at 90 days. Safety outcomes included asymptomatic intracerebral hemorrhage (ICH) and symptomatic ICH assessed using the National Institute of Neurological Disorders and Stroke (NINDS) study and the Safe Implementation of Thrombolysis in Stroke-Monitoring (SITS-MOST) study. We also compared parenchymal hematomas as safety outcome between the two groups defined by the authors themselves in their research. A total of 16 studies were included in the present meta-analysis. The meta-analysis did not report any significant difference between low-dose and standard-dose r-tPA in terms of mortality, symptomatic intracranial hemorrhage (SICH), asymptomatic ICH, and parenchymal hematomas. However, the favorable outcome was significantly greater in patients receiving a standard dose of r-tPA.

## Introduction and background

Cerebrovascular stroke is the second and seventh leading cause of death and disability worldwide [1]. Stroke is a major burden on healthcare systems, but the clinical outlook has become more promising with better patient care and new therapy options [2]. Intravenous thrombolysis utilizing recombinant tissue plasminogen activator (r-tPA) is used in patients with acute ischemic stroke [3]. Thrombolytic therapy with intravenous alteplase at a dose of 0.9 mg/kg of body weight is effective for acute ischemic stroke patients, despite enhancing the risk of intracerebral hemorrhage [4]. However, after a study showed that a lower dose was as effective as the standard dose and associated with a lower risk of intracerebral hemorrhage, the Japanese drug safety authority approved the use of 0.6 mg/kg of body weight [5].

Registry studies conducted in various parts of Asia have produced inconsistent results regarding the use of alteplase [6-7], but the study in the United States has observed a high risk of symptomatic intracerebral hemorrhage among Asian patients treated with 0.9 mg of alteplase per kilogram [8]. Due to differing perceptions of the risks of intracerebral hemorrhage and varying treatment costs, there have been differences in the doses of intravenous alteplase used to treat acute ischemic stroke patients in Asia [9-10]. The Enhanced Control of Hypertension and Thrombolysis Stroke Study (ENCHANTED) was developed to compare low-dose and standard-dose intravenous alteplase use in patients with acute ischemic stroke. The trial did not show non-inferiority of low-dose alteplase but found a modest reduction in intracranial hemorrhages [11].

A 2017 meta-analysis involving 12 studies found no significant difference between low dose and standard dose of r-tPA in terms of mortality and favorable outcomes [12]. Since this meta-analysis, certain new studies have been conducted to compare the efficacy and safety of low doses and standard doses, including non-randomized studies [13] and retrospective cohorts conducted in different regions of the world [14-16].

An updated meta-analysis of the existing evidence in this area could provide a more definitive answer to this question by pooling the results of multiple studies and conducting a systematic analysis of the data. This would allow researchers to evaluate the overall effectiveness and safety of low-dose thrombolytic therapy compared to standard-dose therapy. The aim of this meta-analysis is to compare the efficacy and safety of low-dose and standard-dose r-tPA in patients with acute ischemic stroke.

## Review

Methodology

The present meta-analysis was conducted according to the Meta-Analysis of Observational Studies in Epidemiology (MOOSE) guidelines.

Literature Search Strategy and Study Selection

We conducted a systematic search in PubMed, Embase, and the Cochrane Library to identify studies published between January 1, 2010, and January 31, 2023, using the following terms: "stroke," "alteplase," "doses," "efficacy," "tissue plasminogen activator," "r-tPA," and "safety." To identify relevant articles for this study, a comprehensive search was conducted using Medical Subject Headings (MeSH) terms and boolean algebra operators to combine and refine search keywords in a systematic and structured manner. We manually searched the reference lists of all included studies for additional relevant records. All identified records were imported into an EndNote Library X9, and duplicates were removed. Initial screening was conducted based on the title and abstract of each study, and full texts of all eligible articles were retrieved. We carried out a detailed assessment of eligibility criteria using predefined inclusion and exclusion criteria. Two authors independently conducted the study search and selection process, and any disagreement was resolved through discussion or consensus with a third investigator. 

Eligibility Criteria

We included all prospective or retrospective studies that met the following criteria: (a) patients with ischemic stroke receiving alteplase, (b) comparison of two doses of alteplase, and (c) reporting of outcomes assessed in the present analysis. We included only those studies in which patients were presented within 4.5 hours of the onset of stroke. Lastly, we included those studies that were published in the English language only. We defined the standard dose of alteplase as >0.85 mg per kilogram of body weight, while any dose other than the standard dose was regarded as a low dose. We excluded reviews, cross-sectional studies, case reports, and case series.

Outcomes

Primary efficacy outcomes included favorable outcomes (Modified Rankin Scale scores of 0-2), while secondary efficacy outcome was all-cause mortality at 90 days. Safety outcomes included asymptomatic intracerebral hemorrhage (ICH) and symptomatic ICH assessed using the National Institute of Neurological Disorders and Stroke (NINDS) study and the Safe Implementation of Thrombolysis in Stroke-Monitoring (SITS-MOST) study at 90 days. We also compared parenchymal hematomas as safety outcome between the two groups defined by the authors themselves in their research.

Data Extraction

Data extraction was performed using a data collection sheet developed in Microsoft Excel. The data extracted from the included studies included author name, year of publication, study design, dose, sample size, participants' characteristics, and outcome measures. One investigator extracted the data, and the other author cross-checked it and entered it into RevMan software for data analysis.

Data Analysis

Data analysis was done using RevMan Version 5.4.1 (The Cochrane Collaboration, United Kingdom). A risk ratio (RR) with 95% confidence intervals (CIs) was calculated for outcome variables. P-values less than 0.05 were considered significant. A random effect model was used to pool studies with significant heterogeneity as determined by the I-square value of more than 50%. We performed subgroup analysis based on the continent where the study was conducted and also on study design (retrospective and prospective studies). 

Results

**Figure 1:**
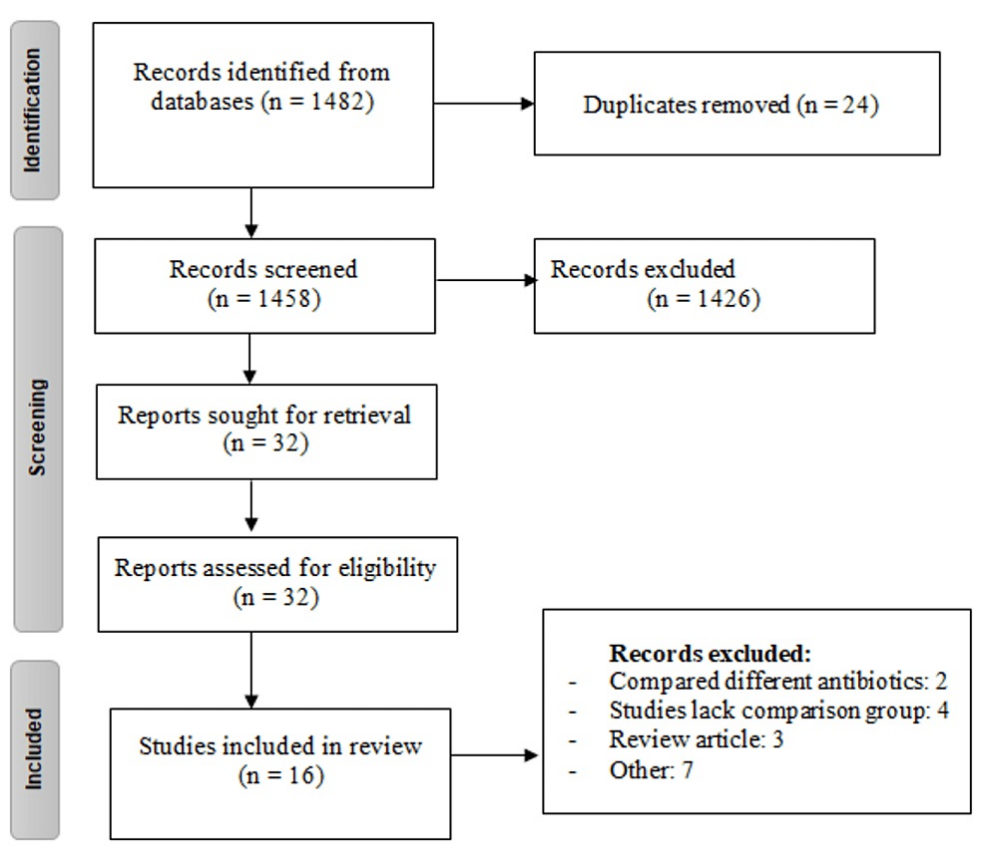
Flowchart showing the study selection process

One thousand four hundred eighty-two studies were initially retrieved from Embase and PubMed. The majority of records were excluded upon titles or abstracts screening. Full texts of all eligible studies were obtained and assessed for detailed assessment of inclusion and exclusion criteria. After a full-text review of 32 papers, 16 were included in the present meta-analysis. Figure 1 shows the flowchart of the selection of studies. Table 1 shows the characteristics of included studies. Among all included studies, 12 were retrospective cohort studies, while one randomized control trial (RCT) was included. There was a significant between-group difference in age in four studies and gender in three studies. However, no significant difference in the history of stroke was found between the two groups in any of the included studies.* *

**Table 1:** Characteristics of included studies RCT - randomized control trial; NR - not reported *significantly different in the two groups

Author	Year	Design	Region	Groups	Dose	Sample size	Age (mean years)	Female (%)	Previous stroke history (%)
Anderson et al. [11]	2016	RCT	Asia, Europe and South America	Low dose	0.6 mg	1654	68 vs 67	38.3 vs 37.4	17.4 vs 18.4
				Standard dose	0.9 mg	1643			
Aulickey et al. [17]	2013	Retrospective cohort	Europe	Low dose	<0.85 mg	62	71 vs 69	50 vs 42	24 vs 19
				Standard dose	0.85-0.95 mg	171			
Chao et al. [18]	2019	Prospective cohort	Asia	Low dose	0.6 mg	108	83 vs 82	54.6 vs 46.8	NR
				Standard dose	0.9 mg	141			
Chen et al. [19]	2012	Retrospective cohort	Asia	Low dose	0.7 mg	105	67.9 vs 67.9	38.1 vs 34.6	16.2 vs 17.3
				Standard dose	0.9 mg	156			
Chen et al. [13]	2022	Non-randomized trial	Asia	Low dose	0.6 mg	192	72 vs 63*	33.3 vs 33.4	22.4 vs 17.6
				Standard dose	0.9 mg	182			
Kim et al. [20]	2015	Retrospective cohort	Asia	Low dose	0.6 mg	450	69 vs 68.2	45.6 vs 38.7*	NR
				Standard dose	0.9 mg	1076			
Liao et al. [21]	2014	Retrospective cohort	Asia	Low dose	0.5-0.6 mg	131	68 VS 63	43.5 vs 37.8	16.03 vs 13.27
				Standard dose	0.85-0.95 mg	678			
Liu et al. [22]	2019	Retrospective cohort	Asia	Low dose	0.6 mg	60	70 vs 71	25 vs 37.2*	30 vs 20.85
				Standard dose	0.85-0.9 mg	494			
Mai et al. [15]	2021	Retrospective cohort	Asia	Low dose	0.6 mg	73	62.4 vs 63.6	37 vs 38.2	5.5 vs 0
				Standard dose	0.9 mg	34			
Sadeghi-Hokmabadi et al. [14]	2021	Retrospective cohort	Asia	Low dose	0.6 mg	149	77 vs 68*	51.6 vs 42.2	0 vs 2.3
				Standard dose	0.9 mg	906			
Salem et al. [16]	2021	Prospective cohort	Asia	Low dose	0.6 mg	40	60.6 vs 61	45 vs 75	12.5 vs 10
				Standard dose	0.9 mg	40			
Sharma et al. [23]	2010	Retrospective cohort	Asia	Low dose	0.6 mg	48	NR	NR	NR
				Standard dose	0.9 mg	82			
Skrbic et al. [24]	2019	Retrospective cohort	Europe	Low dose	0.6 mg	45	67.5 vs 61.9*	55.6 vs 46.1	NR
				Standard dose	0.9 mg	165			
Wang et al. [25]	2019	Retrospective cohort	Asia	Low dose	0.6 mg	1540	68.4 vs 65.1*	39 vs 35.7*	20.6 vs 18.5
				Standard dose	0.9 mg	4360			
Yang et al. [26]	2016	Retrospective cohort	Asia	Low dose	0.6 mg	46	65.5 vs 64.5	43.5 vs 45.2	NR
				Standard dose	0.9 mg	62			
Zhou et al. [27]	2010	Retrospective cohort	Asia	Low dose	0.6-0.8 mg	54	71.4 vs 72.7	33.33 vs 37.3	NR
				Standard dose	0.9 mg	51			

Efficacy Outcomes

**Figure 2:**
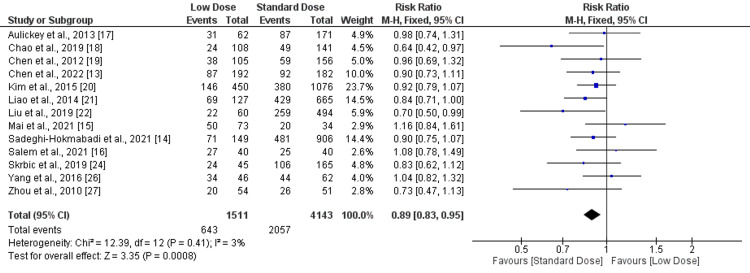
Forest plot comparing favorable outcome between low-dose and standard-dose recombinant tissue plasminogen activator (r-tPA) Sources: [13-22,24,26-27]

Favorable outcome: thirteen studies reported favorable outcomes, and 10 of these studies showed fewer favorable outcomes in patients receiving the low dose of alteplase compared to the standard dose. Three studies reached statistical significance [18,22]. Pooled estimates showed that favorable outcomes were significantly higher in patients receiving the standard dose compared to the low dose (RR: 0.89, 95% CI: 0.83-0.95), as shown in Figure 2. Low heterogeneity was reported among the study results (I-square: 0%).

**Figure 3:**
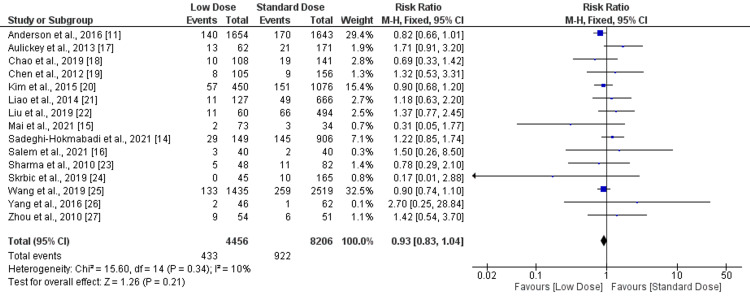
Forest plot comparing mortality between low-dose and standard-dose recombinant tissue plasminogen activator (r-tPA) Sources: [11,14-27]

Mortality: in the present meta-analysis, a total of 15 studies were included that compared the risk of mortality between low-dose and standard-dose alteplase. The incidence of mortality was lower in the low-dose group (9.72%) compared to the high-dose group (11.24%; RR: 0.93, 95% CI: 0.83-1.04), as shown in Figure 3. No significant heterogeneity was reported among the study results (I-square: 10%).

Safety Outcomes

**Figure 4:**
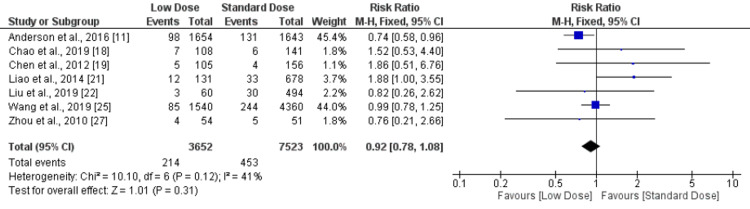
Forest plot comparing SICH (NINDS) between low-dose and standard-dose recombinant tissue plasminogen activator (r-tPA) SICH - symptomatic intracranial hemorrhage; NINDS - National Institute of Neurological Disorders and Stroke Sources: [11,18-19,21-22,25,27]

**Figure 5:**
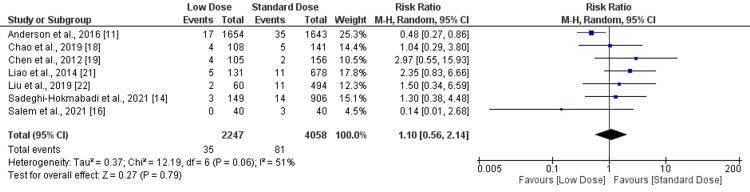
Forest plot comparing SICH (SITS-MOST) between low-dose and standard-dose recombinant tissue plasminogen activator (r-tPA) SICH - symptomatic intracranial hemorrhage; SITS-MOST - Safe Implementation of Thrombolysis in Stroke-Monitoring Study Sources: [11,14,16,18-19,21-22]

Symptomatic intracranial hemorrhage (SICH): SICH was identified in seven studies according to the NINDS definition and seven studies according to the SITS-MOST definition. Therefore, pooled analyses for SICH were conducted separately based on NINDS and SITS-MOST definitions. There was no significant difference in SICH using the NINDS definition between low dose and standard dose (RR: 0.92, 95% CI: 0.78-1.08, I-square: 41%), as shown in Figure 4. Moreover, we did not observe any significant difference in SICH using the SITS-MOST definition between low dose and standard dose (RR: 1.10, 95% CI: 0.56-2.14, I-square: 51%) as shown in Figure 5.

**Figure 6:**
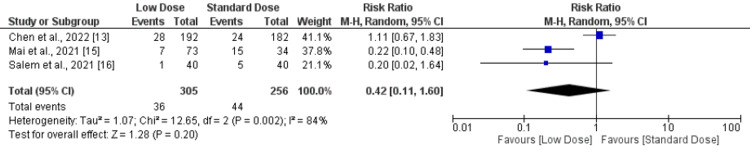
Forest plot comparing asymptomatic ICH between low-dose and standard-dose recombinant tissue plasminogen activator (r-tPA) ICH - intracerebral hemorrhage Sources: [13,15-16]

**Figure 7:**
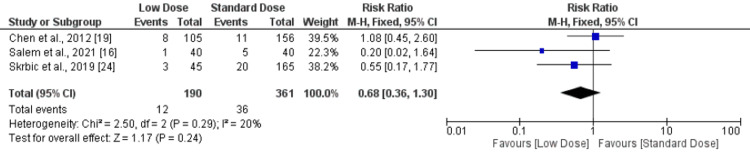
Forest plot comparing parenchymal hematomas between low-dose and standard-dose recombinant tissue plasminogen activator (r-tPA) Sources: [16,19,24]

Non-symptomatic ICH and parenchymal hematomas: the present meta-analysis revealed no significant difference in non-symptomatic ICH and parenchymal hematomas between standard- and low-dose IV-tPA patients, as shown in Figures 6 and 7, respectively.

Subgroup Analysis

When combining only studies conducted in Asia, the pooled estimate for all-cause mortality showed no heterogeneity (I-square: 0%), and no statistical difference was found between the two groups, as shown in Table 2. For favorable outcomes, the results were consistent with the pooled analysis for the Asian population.

**Table 2:** Results of subgroup analysis RR - risk ratio; CI - confidence interval; SICH - symptomatic intracranial hemorrhage; SITS-MOST - Safe Implementation of Thrombolysis in Stroke-Monitoring Study; NINDS - National Institute of Neurological Disorders and Stroke *significant at p-value <0.05

Groups	Outcomes	RR (95% CI)	I-square
Asia	Mortality	0.97 (0.85-1.10)	0%
	Favorable outcome	0.89 (0.82-0.95)*	15%
	SICH (SITS-MOST)	1.54 (0.87-2.71)	0%
	SICH (NINDS)	1.07 (0.87-1.32)	2%
Prospective	Mortality	0.81 (0.66-1.00)	0%
	Favorable outcome	0.86 (0.73-1.01)	50%
	SICH (SITS-MOST)	0.53 (0.31-0.88)*	0%
	SICH (NINDS)	0.77 (0.60-0.99)*	40%
Retrospective	Mortality	0.99 (0.87-1.13)	8%
	Favorable outcome	0.89 (0.83-0.97)*	0%
	SICH (SITS-MOST)	1.90 (0.99-3.63)	0%
	SICH (NINDS)	1.05 (0.85-1.30)	15%

Based on the study design, the pooled analysis of prospective studies showed a significant reduction of SICH (using definitions of SITS-MOST and NINDS) in the low-dose tPA group compared to the standard group. However, the pooled analysis of retrospective studies did not find any significant difference between the two groups in terms of SICH, as shown in Table 2. We also performed a subgroup analysis on favorable outcomes. We observed a significant impact of the standard dose on favorable outcomes in the pooled analysis of retrospective studies. On the contrary, in the pooled analysis of prospective studies, individuals with favorable outcomes were greater in the standard group compared to the low-dose group, but the difference was statistically insignificant.

Discussion

The present meta-analysis assessed the differences in safety and efficacy between low-dose and standard-dose intravenous r-tPA therapy in patients with ischemic stroke. The meta-analysis found that patients receiving standard-dose r-tPA had significantly greater favorable outcomes compared to those receiving low-dose r-tPA. A past meta-analysis of 11 studies conducted by Tan et al. [12] found no significant difference in favorable outcomes between low-dose and standard-dose alteplase using a random-effects model. However, the majority of the studies included in the present meta-analysis showed a beneficial impact of standard-dose on favorable outcomes [13-14,17-22,24,26-27]. Two out of the ten studies that showed a beneficial impact of standard-dose on favorable outcomes were significant [18,21]. We conducted a subgroup analysis based on the continent in which the study was carried out. Among all the included studies, 14 were exclusively conducted in Asia [13-16,18-23,25-27]. The favorable outcome was significantly higher in patients receiving standard-dose of r-tPA.

The findings of the present meta-analysis suggest that there is a connection between using a standard dose of r-tPA and an increased likelihood of a favorable outcome. However, it is important to consider that most of the studies used in this analysis were not randomized, which means that there may have been selection bias - patients who received a lower dose may have had a lower chance of a positive outcome. This is because patients who are at a higher risk of bleeding on admission are often given a lower dose of rtPA, and these patients tend to have a larger area of brain damage or other factors that can reduce the likelihood of a favorable outcome at three months. Therefore, the potential benefits of a standard dose of rtPA on positive outcomes at three months should be confirmed by further randomized controlled trials in the future.

The study conducted by Liu et al. [28] found no significant difference between the two groups in the incidence of SICH using a random-effects model. As there were different definitions of SICH among the various studies, the reliability of the results may be challenged. In the present meta-analysis, we conducted a pooled estimate of each definition of SICH, including SITS-MOST and NINDS. When using the definition of SITS-MOST and NINDS, we did not find any significant difference in the incidence of SICH. Only the trial conducted by Anderson et al. showed that the incidence of SICH was significantly higher in patients randomized to the low-dose group compared to the standard dose as per the definition of NINDS and SITS-MOST [11]. Moreover, the incidence of parenchymal hematomas and asymptomatic ICH was higher in the standard-dose group compared to low-dose alteplase, but the differences were statistically insignificant.

Although developing countries account for 85% of stroke cases globally, the administration of IV-tPA to patients in these countries is quite low [29]. One of the primary reasons for this is financial constraints, with the cost of standard-dose IV-tPA per patient being $1400 (USD), which is a significant burden for most patients [30]. This is particularly evident in Iran, where only 30% of stroke patients are able to afford IV-tPA out of their own pockets [31]. Research conducted in northwest India revealed that out of 22 eligible patients for thrombolysis, only five were administered with IV-tPA, as the remaining patients could not afford the expensive treatment [32]. However, if randomized controlled studies confirm the effectiveness and safety of low-dose IV-tPA, the cost of the medicine could be lowered and ultimately reduce the financial burden experienced by potential patients [28].

The current meta-analysis showed that the standard dose was still the best option for patients with acute ischemic stroke. Regarding individual patients, who might get benefit from a lower dosage? A study involving 3479 patients with acute ischemic stroke found that for those with a moderate stroke (NIHSS 5-14), lower amounts of tPA were linked to a decrease in severe bleeding in the brain and had similar effectiveness to higher doses [33]. Wang et al. also showed that a low dose of tPA may be preferable in patients of younger age. Mild neurological impairment and lower systolic blood pressure [25]. 

It is important to note that while the present meta-analysis aimed to provide a more comprehensive estimate of SICH, there may still be some heterogeneity across the included studies that could affect the overall results. Further studies with larger sample sizes and standardized definitions of SICH are needed to provide more conclusive evidence. Additionally, the potential clinical significance of the observed differences in SICH incidence and other hemorrhagic complications between low and standard-dose alteplase warrants careful consideration when making treatment decisions for individual patients. Future research may include a diverse population to assess the impact of tPA on these individuals.

Study Limitations

The present meta-analysis has certain limitations. Firstly, the low dose of alteplase in various included studies ranged from 0.5 to 0.95 mg per kg of body weight. Secondly, the majority of included cohort studies were performed in the Asian population. Therefore, the findings need to be interpreted with caution in the overall world population because of genetic and racial differences. However, we have conducted a subgroup analysis based on continents. Thirdly, only one randomized control trial has been included in the present meta-analysis. Non-randomized designs of studies would cause a selection bias, which may challenge the reliability of outcomes. We did not perform subgroup analysis for randomized trials because of a lack of a number of studies. However, we performed a subgroup analysis based on the prospective and retrospective nature of the studies. Lastly, we did not carry analysis based on the severity of the stroke due to a lack of individual patient data. 

## Conclusions

The present meta-analysis found that compared to a low dose, the standard dose of tPA was effective in enhancing favorable outcomes in patients with acute ischemic stroke without significantly increasing the risk of symptomatic ICH, asymptomatic ICH, and parenchymal hematomas. However, only one randomized trial was included in the present meta-analysis. Given the possibility that efficacy is optimized using the standard of 0.9 mg/ kg tPA, a randomized dose-comparison study is an important research priority to establish the optimal tPA dose in stroke patients. For this reason, RCTs are important to conduct in patients from various backgrounds and stroke severity to determine the optimum dose of tPA. Healthcare professionals need to remain cautious in clinical practice when considering low-dose tPA treatment of acute ischemic patients.
